# Intravenous Tolvaptan Sodium Phosphate Can Fail to Improve Fluid Overload Due to Heart Failure in Patients With Chronic Kidney Disease: A Case Report

**DOI:** 10.1002/prp2.70252

**Published:** 2026-04-16

**Authors:** Takaya Uno, Ichiro Nakakura, Akari Ikeda, Atsuki Hosoda, Yutaro Mukai, Kaori Yamanishi, Naohiro Ohara, Kaori Imanishi, Yoshiko Une, Satoshi Yokoyama, Kouichi Hosomi

**Affiliations:** ^1^ Division of Drug Informatics, School of Pharmacy Kindai University Higashi‐Osaka Osaka Japan; ^2^ Department of Pharmacy National Cerebral and Cardiovascular Center Suita Osaka Japan

**Keywords:** chronic kidney disease, heart failure, tolvaptan, tolvaptan sodium phosphate

## Abstract

Tolvaptan is widely used as an adjunct oral diuretic to loop and thiazide diuretics for the management of fluid overload in patients with congestive heart failure. Recently, tolvaptan sodium phosphate (TSP), a water‐soluble prodrug of tolvaptan, was developed for intravenous administration and has demonstrated efficacy and safety in clinical settings. However, cases of TSP ineffectiveness have not yet been reported. Because oral tolvaptan does not work in some patients, TSP may also be ineffective in certain cases. We report the case of a 74‐year‐old male patient with chronic heart failure and chronic kidney disease who was unresponsive to TSP. The patient underwent off‐pump coronary artery bypass grafting. Initially, intravenous furosemide and oral tolvaptan were administered to manage residual fluid overload; however, intravenous furosemide was discontinued owing to a decline in serum potassium levels. Oral loop and thiazide diuretics in combination with TSP were then added to ongoing oral tolvaptan therapy, but this regimen did not achieve sufficient weight reduction. Subsequently, high‐dose intravenous furosemide was reintroduced in place of oral loop diuretics, with careful monitoring for hypokalaemia. Although high‐dose intravenous furosemide worsened renal dysfunction, it successfully improved fluid overload and reduced plasma B‐type natriuretic peptide levels, effects that persisted even after discontinuation of TSP. This case suggests that in patients with chronic kidney disease who are unresponsive to TSP, high‐dose intravenous furosemide may provide more effective management of fluid retention in heart failure, though renal function must be closely monitored.

## 1 Background

Tolvaptan, an arginine vasopressin type‐2 receptor antagonist, is widely used as an adjunct oral diuretic to loop and thiazide diuretics for the management of fluid overload in patients with congestive heart failure [1]. Recently, tolvaptan sodium phosphate (TSP), a water‐soluble prodrug of tolvaptan, was developed for intravenous administration [2] and has demonstrated efficacy and safety in clinical settings [3, 4]. However, no cases of TSP ineffectiveness have been reported. Oral tolvaptan has already been documented to be ineffective in some patients [5], and these patients were reported to have poor outcomes, including rehospitalization and prolonged hospital stay [5, 6]. Therefore, as with oral tolvaptan, TSP may also be ineffective in certain patients, potentially leading to poor outcomes. We recently encountered a patient with chronic kidney disease who was unresponsive to TSP. To the best of our knowledge, this is the first report of TSP ineffectiveness.

## 2 Case Presentation

**FIGURE 1:**
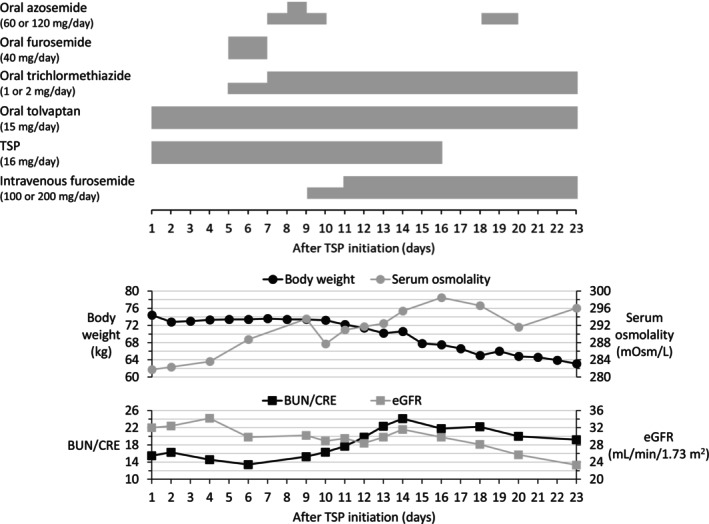
Clinical course of treatment in a patient with congestive heart failure. Days are counted from the initiation of TSP administration. Serum osmolality was estimated using the following formula: Serum osmolality (mOsm/L) = 2 × serum sodium (mEq/L)+serum BUN (mg/dL)/2.8+serum glucose (mg/dL)/18 [7]. Black circles indicate body weight; gray circles indicate estimated serum osmolality; black squares indicate BUN/CRE; gray squares indicate eGFR. BUN, blood urea nitrogen; CRE, creatinine; eGFR, estimated glomerular filtration rate; TSP, tolvaptan sodium phosphate.

A 74‐year‐old male patient with chronic heart failure and chronic kidney disease underwent off‐pump coronary artery bypass grafting. Postoperatively, intravenous furosemide (20 mg/day) and oral tolvaptan (15 mg/day) were initiated to manage residual fluid overload, but intravenous furosemide was discontinued owing to a decline in serum potassium levels. TSP (16 mg/day) was subsequently added to ongoing oral tolvaptan monotherapy to achieve his preoperative target weight of 65 kg (Figure 1).

At the time of TSP initiation, the patient was receiving atorvastatin (20 mg/day), ezetimibe (10 mg/day), vonoprazan (20 mg/day), aspirin (200 mg/day), acetaminophen (1600 mg/day), bisoprolol fumarate (1.25 mg/day), nicorandil (15 mg/day), amlodipine (5 mg/day), *Clostridium butyricum* preparation (60 mg/day), magnesium oxide (990 mg/day), nitroglycerin (27 mg/day), ramelteon (8 mg/day), suvorexant (15 mg/day), linagliptin (5 mg/day), insulin lispro, insulin glargine, and 7.5 g/day of daikenchuto (containing 0.625 g of extract derived from 2.5 g of processed ginger, 1.0 g of Japanese *Zanthoxylum* peel and 1.5 g of ginseng, plus 5.0 g of Koi). Magnesium oxide, ramelteon, and suvorexant doses were adjusted at the patient's discretion, whereas insulin lispro and insulin glargine were adjusted under physician supervision.

Oral diuretics other than oral tolvaptan were introduced between Days 5 and 8 after initiation of TSP (Figure 1). However, these additional oral diuretics increased serum osmolarity without achieving significant weight reduction. On Day 9, intravenous furosemide was introduced instead of oral azosemide. Following intravenous furosemide administration, serum osmolarity and blood urea nitrogen (BUN)/creatinine (CRE) increased, accompanied by weight loss and resolution of edema. On Day 16, TSP was discontinued after the patient's weight returned to near baseline. However, pleural effusion persisted, and the plasma B‐type natriuretic peptide (BNP) level remained elevated (Table 1), necessitating the continuation of intravenous furosemide therapy. By Day 23, renal dysfunction had further deteriorated, but the plasma BNP level had decreased.

**TABLE 1:** Laboratory parameters during the treatment period.

After TSP initiation	ALT (U/L)	AST (U/L)	BNP (pg/mL)	BUN (mg/dL)	CRE (mg/dL)	Glucose (mg/dL)	Potassium (mEq/L)	Sodium (mEq/L)
Day 1	11	22	—	26	1.68	152	4.6	132
Day 2	10	19	—	27	1.66	84	4.5	134
Day 4	10	17	—	23	1.58	61	4.5	136
Day 6	12	19	—	24	1.79	75	4.5	138
Day 9	13	18	—	27	1.77	106	4.2	139
Day 10	12	21	669.5	30	1.84	90	4.1	136
Day 11	11	17	650.1	32	1.81	100	3.5	137
Day 12	9	15	—	37	1.87	117	3.3	136
Day 13	9	16	—	40	1.79	111	3.5	136
Day 14	10	17	—	41	1.70	121	3.5	137
Day 16	11	21	766.3	39	1.79	118	3.6	139
Day 18	10	16	—	42	1.89	137	3.6	137
Day 20	13	18	—	41	2.05	125	3.8	135
Day 23	13	20	383.8	43	2.24	120	3.8	137

Abbreviations: ALT, alanine aminotransferase; AST, aspartate aminotransferase; BNP, B‐type natriuretic peptide; BUN, blood urea nitrogen; CRE, creatinine; TSP, tolvaptan sodium phosphate.

Laboratory parameters and non‐diuretic therapeutic interventions during the treatment period (Days 1–23) are summarized in Tables 1 and 2, respectively. The patient reported no symptoms of thirst or dry mouth. No cytochrome P450 (CYP)–inducing agents, which could affect tolvaptan metabolism, were co‐administered.

**TABLE 2:** Non‐diuretic therapeutic interventions during the treatment period.

After TSP initiation	Intervention
Day 10	*Clostridium butyricum* preparation and daikenchuto were discontinued. Edoxaban (30 mg/day) was initiated.
Day 11	Potassium chloride (3600 mg/day) was initiated.
Day 17	Acetaminophen was discontinued.

Abbreviation: TSP, tolvaptan sodium phosphate.

## 3 Discussion

In this case, despite the administration of oral tolvaptan and TSP, additional oral loop and thiazide diuretics were required, which led to an increase in serum osmolality. This finding suggests that absorption of oral loop and thiazide diuretics was preserved. In contrast, tolvaptan monotherapy, including oral tolvaptan and TSP without concomitant diuretics other than tolvaptan, did not sufficiently increase serum osmolality or reduce body weight. A previous report indicated that tolvaptan monotherapy, even without loop diuretics, could reduce body weight [8]. Therefore, our findings suggest that in this patient, both oral tolvaptan and intravenous TSP were ineffective, regardless of the route of administration. As tolvaptan is primarily metabolized by CYP3A, co‐administration of CYP3A inducers can reduce drug exposure [9]. However, in this case, no CYP3A inducers were administered during the study period, making reduced exposure an unlikely cause. Thus, the lack of therapeutic response may be related to pharmacodynamic rather than pharmacokinetic factors, such as absorption and metabolism.

One key pharmacodynamic factor associated with tolvaptan activity is aquaporin‐2, which is expressed in response to arginine vasopressin stimulation and translocated to the surface of the collecting duct, where it facilitates water reabsorption. Tolvaptan exerts its effect by antagonizing the vasopressin type‐2 receptor, thereby inhibiting aquaporin‐2–mediated water reabsorption [10]. For tolvaptan to be effective, the cascade from arginine vasopressin to aquaporin‐2 expression must remain intact. Several reports have shown that aquaporin‐2 is not expressed in renal tissue from patients with diabetic nephropathy who are unresponsive to tolvaptan [11, 12]. In the present case, the patient had renal dysfunction, and reduced aquaporin‐2 expression secondary to impaired renal function may have contributed to the inadequate response. Meanwhile, high‐dose intravenous furosemide worsened renal dysfunction but achieved sufficient weight loss, increased serum osmolality and BUN/CRE (an indicator of dehydration), and decreased plasma BNP levels. Importantly, these beneficial effects persisted even after discontinuation of TSP. These findings further suggest that tolvaptan unresponsiveness in this case was likely due to pharmacodynamic factors, such as reduced aquaporin‐2 expression.

This case suggests that patients with chronic kidney disease were unresponsive to TSP. In such clinical scenarios, alternative approaches, including high‐dose furosemide, may provide more effective management of fluid overload in heart failure, provided that renal function is closely monitored. Nevertheless, as this report describes a single patient, the potential influence of confounding factors—such as concomitant medications and postoperative changes—cannot be excluded, and a definitive causal relationship cannot be established. Accordingly, further confirmation through larger and well‐designed prospective studies is warranted to validate these findings.

## Author Contributions

**Takaya Uno:** conceptualization, data curation, funding acquisition, investigation, writing – original draft, writing – review and editing. **Ichiro Nakakura:** investigation, project administration, writing – review and editing. **Akari Ikeda:** writing – review and editing. **Atsuki Hosoda:** writing – review and editing. **Yutaro Mukai:** writing – review and editing. **Kaori Yamanishi:** writing – review and editing. **Naohiro Ohara:** writing – review and editing. **Kaori Imanishi:** writing – review and editing. **Yoshiko Une:** supervision, writing – review and editing. **Satoshi Yokoyama:** writing – review and editing. **Kouichi Hosomi:** writing – review and editing.

## Funding

This work was supported by the OSAKA‐YAKUGYO‐CLUB, fiscal year 2022 (Grant 5).

## Ethics Statement

This case report forms part of a study approved by the Ethics Committee of Kindai University School of Pharmacy (approval no. 25–267) and the Ethics Committee of the National Cerebral and Cardiovascular Center (approval no. R23018‐6).

## Consent

This study was a retrospective observational study, and informed consent for participation was obtained via the opt‐out method.

## Conflicts of Interest

The authors declare no conflicts of interest.

## Data Availability

Due to the nature of this research, the data obtained during the study cannot be shared openly.
